# Is positive school climate associated with better adolescent mental health? Longitudinal study of young people in England

**DOI:** 10.1016/j.ssmmh.2021.100033

**Published:** 2021-12

**Authors:** Baptiste Leurent, Matthew Dodd, Elizabeth Allen, Russell Viner, Stephen Scott, Chris Bonell

**Affiliations:** a Department of Medical Statistics, London School of Hygiene and Tropical Medicine, Keppel Street, London, WC1E 7HT, UK; b Institute of Child Health, University College London, 30 Guilford St, London, WC1N 1EH, UK; c Institute of Psychiatry, Psychology and Neuroscience, King's College London, 16 De Crespigny Park, London, SE5 8AF, UK; d Department of Public Health, Environments and Society, London School of Hygiene and Tropical Medicine, UK

**Keywords:** Schools, Adolescents, Mental wellbeing, Psychological problems, School engagement

## Abstract

**Background and objectives:**

Studies suggest that individual student-reported connection to school is associated with better mental health. However, there is less evidence for associations between schools’ overall school climate and the mental health of their students. This may reflect limitations in which mental health outcomes have been examined. We conducted a large longitudinal study in schools, hypothesising that we would find associations at both the student and school levels between student-reported positive school climate, and reduced student conduct and emotional problems and improved mental wellbeing.

**Methods:**

We tracked students in 20 English secondary schools from near the end of the first year of secondary school (age 11/12) over 3 years using reliable measures of school climate and mental health.

**Results:**

We found associations between student-level reports of positive school climate at baseline, and reduced conduct and emotional problems and better mental wellbeing at 3-year follow-up adjusting for various potential confounders. We also found some evidence of adjusted associations between baseline school-level measures of overall positive climate and better student mental health at follow-up. However, these student- and school-level associations reduced considerably when also adjusting for baseline mental health.

**Conclusions:**

Our findings suggest that there are associations between school climate and student mental health at both the student and school level but these associations are complex and not necessarily causal.

## Highlights

• Previous studies provide little evidence that mental health differ between schools or is influenced by school climate.
• These studies' ability to identify school effects may have been undermined by choice of outcomes.
• Our study suggests that mental health may be better in schools with more student belonging.

## 1 Introduction

Whether school climate is influential on adolescent mental health is an important question because mental health problems are a significant cause of disability and commonly arise in adolescence. Mental illness is the largest cause of disability in the UK (England Mental Health Taskforce, 2016). Studies report that most mental disorders manifest before age 25 years, often between 11 and 18 years (Kessler et al., 2005). The NHS Digital Survey found that, among those aged 5–19, 13% have at least one mental health disorder (Sadler et al., 2018). There is evidence that some adolescent mental health problems, such as mood and emotional disorders, self-harm and suicidality, have become more prevalent in recent years (Twenge et al., 2019; Vizard et al., 2020). Mental disorders during adolescence have important negative consequences for young people's education, employment, relationships and adult mental health (Patton et al., 2018).

Existing theory suggests that a positive school climate can promote adolescent mental health (Bonell et al., 2013a; Markham and Aveyard P, 2003). This theory suggests that, in schools with climates characterised by good relationships between teachers and students, student participation in school decisions and teaching which engages student interest, students are more likely to engage both educationally in learning and in the sense of feeling a sense of belonging to the school community. These are theorised to enable each student develop the emotional and social skills and the positive relationships need to help build and protect their mental health. The theory also suggests that school climate may enhance student mental health via another pathway involving ‘herd effects’ whereby students in school with more positive climates are surrounded by other students who are more engaged and themselves have stronger social and emotional skills and supportive social relationships.

However, this theorisation of positive school climate generating mental health benefits is not currently supported by empirical evidence. Although there is consistent evidence that, at an individual level, student connection to school and educational engagement are associated with better mental health (Aldridge & McChesney, 2018; Kidger et al., 2012), there are few studies and less evidence that adolescent mental health varies between schools or is associated with school-level measures of positive school climate (Kidger et al., 2012). This contrasts with the evidence for other areas of health, where there is consistent evidence for associations between positive school-level climate and lower rates of student smoking, alcohol and drug use, violence and sexual risk behaviours (Bonell et al., 2013b; Coulter et al., 2016; Peterson et al., 2020). It may be that the associations between school climate and anti-social behaviours, such as violence and smoking, really are stronger than are associations between school climate and mental health outcomes perhaps because these outcomes are affected by but can also affect the school climate. However, it may instead be that mental health outcomes are just as strongly associated with, and causally related to, school climate but that existing studies have failed to detect such associations.

It is plausible that schools do influence mental health. Young people spend the greatest proportion of their waking hours in school (Rutter et al., 1979). Schools have the potential to support mental health via positive friendships and learning new skills, which tend to improve mental health (Prati et al., 2018) as well as via schools enabling learning of social skills and emotional self-management (Durlak et al., 2011). Schools will also vary in the extent to which students have negative experiences, such as bullying and educational disengagement, which can harm mental health (Modin et al., 2018).

However, a systematic review by Kidger et al. (2012) found five studies examining associations between school-level exposures and student mental health, finding little evidence of such associations (Kidger et al., 2012). One longitudinal study of Australian adolescents included in this review reported an association between school attended and student depression but with much greater variance between students than between schools (Roeger et al., 2001). Also included in this review, two large US longitudinal studies of high school students found no associations between school-level exposures and mental health outcomes: one of these studies finding no association between a school-level measure of positive climate and negative mental health (depression, anger or negative coping) (Cook et al., 2002), and the other study finding no association between a measure of school-level supportive environment and suicidal behaviours (Winfree & Jiang, 2010). Two longitudinal US studies, also included in the review, reported associations contrary to those hypothesised. The first reported higher rates of adolescent depression and suicide in small compared with large schools (whereas the study authors had hypothesised that small schools would support better mental health because they are characterised by more dense networks of supportive relationships) (Terling Watt, 2003). The second reported that increased student depression was associated with a supportive school environment (Kasen et al., 1990). Overall, the review found stronger evidence for student mental health being associated with individual-level than school-level educational exposures (Kidger et al., 2012).

More recently, a Scottish study tracking individuals from school into middle age found no association between school attended and adult self-rated mental health (Dundas et al., 2014). Another study reported that young people from sexual minorities living in US states and cities where schools had school climates characterised by safe spaces and gay-straight alliances self-reported lower rates of suicidal thoughts. However, this research did not explore more general measures of mental health or examine school-level influences (Hatzenbuehler et al., 2014).

In contrast, there is some evidence that interventions which aim to modify the school social environment, building supportive relationships and student participation, can improve student mental wellbeing, and reducing conduct and emotional problems (Bonell et al., 2013c, 2018; O'Reilly et al., 2018; Shinde et al., 2018).

The lack of consistent evidence for associations between school-level climate and mental health might be explained by limitations in existing observational studies. Few studies have examined the mental health outcomes most plausibly impacted by school social environment, such as conduct and emotional problems and mental wellbeing (Bonell et al., 2019a). Variation in such outcomes might be more likely to be explained by current environment factors than might be the case with outcomes such as depression and anxiety with more variance explained genetic factors and early childhood environments (Franic et al., 2010). It may also be that some of the outcomes examined in previous studies, including depression and suicidality, are less prevalent than outcomes such as conduct problems, so that studies have lacked the statistical power to examine associations with school climate. Previous studies have not used established measures of school climate. As a result, exposure misclassification might be another factor in their not reporting associations between this and mental health outcomes.

To attempt to address these limitations in the existing evidence, we examined the association between student- and school-level measures of positive school climate and adolescent mental health, drawing on a large longitudinal study data tracking students from age 11/12 years (when students were nearing the end of their first year at secondary school) to age 14/15 years in English secondary schools (Bonell et al., 2018). This study assessed student-reported school climate (in terms of good student-teacher relationships, sense of school belonging, student participation in decisions and commitment to learning) at baseline, providing a student-level measure of positive climate and also an aggregated school-level measure (calculated from the mean across students). The study measured student self-reported mental wellbeing, and conduct and emotional problems at baseline and at follow-up 36 months later. Hence it was possible to examine the temporality of any associations between school climate and changes in our outcomes while adjusted for potential confounding from socio-demographic factors and baseline mental health.

This paper aimed to test two main hypotheses. Firstly, we hypothesised that, at the individual level, student-reported school climate at baseline would be positively associated with decreased conduct and emotional problems and increased mental wellbeing at follow-up. Secondly, we hypothesised that there would be an adjusted association at the school level between positive overall school climate at baseline and better mental health at follow-up independent of any individual-level association between student-reported climate and mental health.

## 2 Materials and methods

### 2.1 Design

Our study involved a secondary analysis of data from the control arm of the INCLUSIVE randomised controlled trial of a whole-school bullying prevention intervention (Bonell et al., 2018). We restricted analysis to the control arm because: the intervention in question was effective in promoting various measures of mental health including reducing conduct and emotional problems and improving mental wellbeing so it would not be appropriate to analyse both arms together. We did not think it worthwhile to report a separate analysis focused on the intervention arm because the generalisability of any findings would be extremely limited given that the intervention is currently scaled up in very few schools.

The trial was registered with the ISRCTN registry (10751359). Forty secondary schools, representative of those in south-east England, were randomly allocated after baseline surveys 1:1 to intervention or control (usual treatment) arms (mean 196 students per school; range 102–257). The intervention and the trial ran for 36 months. All students deemed competent by their teachers to decide whether to give informed consent for participation were invited to complete surveys at baseline (age 11–12) and at 24- and 36-month follow-up. Students consenting to participate and not withdrawn by parents completed paper questionnaires in classes in private, supported by trained researchers, with teachers present but unable to read responses. The trial was approved by University College London ethics committee (ref. 5248/001). Full methods are reported elsewhere (Bonell et al., 2018). Our secondary analysis draws on baseline and 36-month follow-up data. Reporting aligns with STROBE guidelines (von Elim et al., 2007).

### 2.2 Measures

We assessed school climate at baseline as our exposure, drawing on the Beyond Blue School Climate Questionnaire (BBSCQ) student self-report measure (Table 1). This scale involves four subscales assessing relationships with teachers, sense of belonging in the school community, student participation in school decisions and student commitment to learning. Each subscale was based on 4–10 items (Table 1). The subscale score was calculated as the average of these items. The overall BBSCQ score was calculated as the average of the four subscales with higher scores indicating a positive assessment of climate. This measure was developed in Australia (Sawyer et al., 2010) using questions from the Gatehouse (Bond et al., 2004), Quality of School Life (Epstein & McPartland, 1976), Patterns of Adaptive Learning (Roeser et al., 1996), Manitoba School Improvement Survey (Earl et al., 2003), and Psychological Sense of School Membership (Goodenow, 1993) instruments. A Cronbach's alpha for the scale of 0.85 was reported for the original Australian adolescent sample (Bond, 2011) and 0.80 for the present sample (Bonell et al., 2017). Previous analysis suggests this validly measures a health-promoting school climate (Bonell et al., 2019b). Student-level measures of school climate rely on this scale while the school-level measure of school climate was created by averaging student responses to BBSCQ in each school.

**Table 1:** Beyond blue school climate scale.

Subscale	Item	Response
Relationships (10 items)	My teachers are fair in dealing with students	0 (totally disagree) - 4 (totally agree)
	There's at least one teacher or other adult in this school I can talk to if I have a problem	
	I feel I can go to my teacher with the things that are on my mind	
	In this school, teachers believe all students can learn	
	In this school, students' ideas are listened to and valued	
	In this school, teachers and students really trust one another	
	In this school, teachers treat students with respect	
	This school really cares about students as individuals	
	Most of my teachers really listen to what I have to say	
	I really like most of my teachers at this school	
Belonging (8 items)	I feel very different from most other students here	
	I can really be myself at this school	
	Other students in this school take my opinions seriously	
	I am encouraged to express my own views in my class(es)	
	Most of the students in my class(es) enjoy being together	
	Most of the students in my class(es) are kind and helpful	
	Most other students accept me as I am	
	I feel I belong at this school	
Commitment (4 items)	I try hard in school	
	Doing well in school is important to me	
	Continuing or completing my education is important to me	
	I feel like I am successful in this school	
Participation (6 items)	There are lots of chances for students at my school to get involved in sports, clubs and other activities outside class	
	Teachers notice when students are doing a good job and let them know about it	
	At my school, students have a lot of chances to help decide and plan things like school activities, events and policies	
	Student activities at this school offer something for everyone	
	Students have a say in decisions affecting them at this school	
	Students at this school are encouraged to take part in activities, programs and special events	

We assessed student mental health at baseline and at 36 months using the Strengths and Difficulties Questionnaire (SDQ) as a measure of conduct and emotional problems (Goodman, 2006), and the Short Warwick-Edinburgh Mental Wellbeing Scale (SWEMWBS) as a measure of mental wellbeing (Clarke et al., 2011). The SDQ is a brief, validated instrument for detecting behavioural, emotional, and peer problems in children and adolescents, based on 20 four-choices items, with a total problems score ranging 0–40 and higher score indicating higher psychological difficulties. The SWEMWBS is a validated measure of adolescent mental wellbeing based on seven five-option items, and scored 7–35, with higher score indicating higher positive mental wellbeing.

We assessed a number of baseline covariates in addition to baseline mental health: school size; proportion of students entitled to free school meals (as a measure of benefits entitlement among students' families); schools’ local neighbourhood deprivation as measured by the Income Deprivation Affecting Children Index (IDACI) with high scores indicating more deprivation (Department for Education, 2015); student family affluence score (FAS) as a validated measure of socioeconomic status scored 0–9 with high scores indicating greater affluence (Currie et al., 2008); student self-reported biological sex; student self-reported family structure (dichotomised to single/two parent); and student self-reported ethnicity (dichotomised to white British/other).

### 2.3 Analyses

We estimated the associations between our baseline exposures (student- and school-level reported school climate as measured by BBSCQ overall and subscales) and outcomes (student conduct and emotional problems measured by SDQ and mental wellbeing measured by SWEMWBS) at 36-month follow-up. As indicated earlier, the school-level measure of school climate was created by averaging student responses to BBSCQ in each school. We used mixed-effect linear regression including a random-effect by school to take into account the clustered structure of the data. We first examined unadjusted associations, then adjusted for the main covariates (baseline measures of school size, proportion of free school meals, and IDACI score, and student sex, ethnicity, family affluence score and family structure).

Students’ baseline mental health (SDQ and SWEMWBS) was considered as both a potential confounder (because it could be independently associated with baseline school climate and subsequent student mental health) and also potentially on the causal pathway between school climate and subsequent mental health (because mental health at baseline may have been already influenced by the general school climate which students had been experiencing for almost a year before baseline surveys). We therefore ran two adjusted models, one with and one without adjustment for baseline mental health.

We then examined the associations between our outcomes and each of our baseline measures of student- and school-level reported school climate in a further adjusted model including student- and school-level measures of school climate. To better separate the effect from the school-climate to the individual responses, we created a differential student score, defined by the student BBSCQ score minus the school-mean BBSCQ score (on each subscale). We then fitted a regression model including both the school-level BBSCQ, and the differential student score as well as the other covariates.

Given the open-cohort nature of the trial (some students left and some joined the school during follow-up), not all students completed both baseline and 36-month surveys. All students (who completed either and the baseline or 36-month follow-up) were included in the analysis and missing data were imputed. All analyses except descriptive statistics were based on multiply imputed data. Joint modelling multi-level imputation was used to take into account clustering by school. Variables included in the imputation models included the school and students' characteristics, student-reported school climate and the outcomes at baseline and 36 months. A set of 30 imputations was performed and results were combined using Rubin's rules. Given the multiple statistical significance tests conducted, no rigid statistical significance threshold was used but results were interpreted in light of the overall pattern of the estimated associations. All analyses were conducted in Stata v16 (StataCorp, 2019), except the multilevel multiple imputation, which was conducted in R (R Core Team, 2020) using the *jomo* package (Quartagno et al., 2019).

## 3 Results

Surveys were completed by 3347 (92.8%) students at baseline and 3087 (85.0%) at follow-up. Our analysis was based on 3913 students from the 20 control schools with sufficient data for the analyses in question. Of these, 3335 (85.2%) completed the baseline survey, and 3034 (77.5%) the 36 months’ survey (Table 2). Data were available at both time-points for 2481 students (63.4%). There was an equal proportion of male and female students, 42.3% were of white British ethnicity and 72.0% living with two parents. Mean FAS was 6.0 (SD ​= ​1.8). At baseline, the mean BBSCQ overall score was 3.25 (SD ​= ​0.41). At 36-month follow-up, the mean conduct and emotional problems (SDQ) score was 12.2 (SD ​= ​6.1), and mean mental wellbeing (SWEMWBS) was 22.9 (SD ​= ​6.1).

**Table 2:** Baseline characteristics and outcomes at 36 months.

	Number observed			Mean or frequency	(SD or %)
	N	(%)			
**School baseline characteristics (N ​= ​20)**					
School size	20	(100%)		1122	(323)
Proportion of students on free school meals	20	(100%)		0.36	(0.18)
IDACI score	20	(100%)		0.26	(0.20)
BBSCQ relationships with teachers	20	(100%)		3.08	(0.13)
BBSCQ school belonging	20	(100%)		2.96	(0.10)
BBSCQ student participation in decisions	20	(100%)		3.28	(0.11)
BBSCQ commitment to learning	20	(100%)		3.62	(0.05)
BBSCQ Total	20	(100%)		3.25	(0.08)
**Students baseline characteristics (N** = **3913)**					
Sex	3897^a^	(99.6%)			
Male				1937	(49.7%)
Female				1960	(50.3%)
Ethnicity	3899^a^	(99.6%)			
White British				1648	(42.3%)
Other				2251	(57.7%)
Family structure	3318	(84.8%)			
Living with two parents				2388	(72.0%)
Other				930	(28.0%)
Family affluence score	3244	(82.9%)		6.0	(1.8)
BBSCQ relationships with teachers	3326	(85.0%)		3.08	(0.57)
BBSCQ school belonging	3322	(84.9%)		2.97	(0.57)
BBSCQ student participation in decisions	3311	(84.6%)		3.29	(0.54)
BBSCQ commitment to learning	3306	(84.5%)		3.62	(0.43)
BBSCQ Total	3282	(83.9%)		3.25	(0.41)
Conduct & emotional problems	3245	(82.9%)		11.0	(6.0)
Mental wellbeing	3208	(82.0%)		24.1	(5.9)
**Students 36 months outcomes (N** = **3913)**					
Conduct & emotional problems	2989	(76.4%)		12.2	(6.1)
Mental wellbeing	2952	(75.4%)		22.9	(6.1)

SD=Standard Deviation.

IDACI=Income Deprivation Affecting Children Index.

BBSCQ=Beyond Blue School Climate Questionnaire.

a For sex and ethnicity, answers reported at either time-point were used.

In the unadjusted analysis, student-level reported positive school climate at baseline was significantly associated with better student mental health outcomes at follow-up, in terms of both reduced conduct and emotional problems, and improved mental wellbeing, at 36 months (Table 3). This applied to all school climate subscales and the overall measure. For each 1-point increased score on the overall BBSCQ scale, student conduct and emotional problems (SDQ) at follow-up were on average 3.80 point lower (95% CI: −4.87 to −2.71, p ​< ​0.001) and mental wellbeing (SWEMWBS) was 2.97 points higher (95% CI: 2.00 to 3.92, p ​< ​0.001).

**Table 3:** Associations between baseline student-level and school-level measures of school climate and student mental health at 36 months.

Exposure		Outcomes																	
		Conduct & emotional problems									Mental wellbeing								
		Unadjusted analysis			Adjusted analysis^a^			Adjusted analysis^b^			Unadjusted analysis			Adjusted analysis^a^			Adjusted analysis^b^		
		Coeffic.	95% CI	P	Coeffic.	95% CI	P	Coeffic.	95% CI	P	Coeffic.	95% CI	P	Coeffic.	95% CI	P	Coeffic.	95% CI	P
Student level BBSCQ	Teacher relationships	−1.85	−2.47 to −1.22	<0.001	−1.86	−2.47 to −1.27	<0.001	−0.1	−0.58 to 0.37	0.661	1.39	0.79 to 1.98	<0.001	1.43	0.86 to 2.02	<0.001	0.11	−0.42 to 0.64	0.684
	School belonging	−2.61	−3.28 to −1.93	<0.001	−2.53	−3.19 to −1.87	<0.001	−0.35	−0.88 to 0.18	0.194	2.05	1.43 to 2.67	<0.001	2.00	1.41 to 2.61	<0.001	0.41	−0.16 to 0.99	0.158
	Participation in decisions	−1.59	−2.18 to −0.99	<0.001	−1.58	−2.16 to −1.01	<0.001	−0.30	−0.77 to 0.17	0.208	1.41	0.81 to 2.01	<0.001	1.41	0.82 to 2.00	<0.001	0.39	−0.12 to 0.89	0.130
	Commitment to learning	−3.70	−4.70 to −2.72	<0.001	−3.58	−4.55 to −2.61	<0.001	−1.30	−2.06 to −0.55	0.001	2.72	1.87 to 3.57	<0.001	2.71	1.88 to 3.56	<0.001	0.88	0.16 to 1.59	0.017
	Overall	−3.80	−4.87 to −2.71	<0.001	−3.72	−4.77 to −2.68	<0.001	−0.78	−1.57 to 0.02	0.056	2.97	2.00 to 3.92	<0.001	2.97	2.03 to 3.91	<0.001	0.73	−0.08 to 1.54	0.078
School level BBSCQ	Teacher relationships	−2.65	−4.83 to −0.49	0.016	−2.11	−3.79 to −0.44	0.013	−1.04	−2.87 to 0.80	0.267	2.25	−0.31 to 4.83	0.085	1.57	−0.68 to 3.82	0.172	0.89	−1.36 to 3.14	0.439
	School belonging	−3.33	−6.41 to −0.26	0.033	−2.00	−4.19 to 0.18	0.072	−0.82	−3.23 to 1.59	0.505	4.01	0.71 to 7.31	0.017	2.70	−0.07 to 5.48	0.056	1.90	−0.91 to 4.71	0.184
	Participation in decisions	−4.08	−6.41 to −1.76	0.001	−2.31	−4.27 to −0.34	0.022	−1.07	−3.29 to 1.15	0.343	4.73	2.08 to 7.38	<0.001	2.48	−0.12 to 5.08	0.062	1.98	−0.59 to 4.55	0.130
	Commitment to learning	−7.80	−14.4 to −1.22	0.020	3.35	−3.99 to 10.7	0.371	3.70	−3.98 to 11.39	0.345	4.08	−3.94 to 12.1	0.319	−6.12	−15.5 to 3.26	0.200	−4.77	−13.71 to 4.17	0.296
	Overall	−5.59	−8.81 to −2.37	0.001	−3.10	−5.76 to −0.45	0.022	−1.36	−4.33 to 1.62	0.372	5.61	1.74 to 9.48	0.004	2.99	−0.57 to 6.57	0.100	2.05	−1.53 to 5.64	0.262

Based on imputed dataset, N ​= ​3913 for all analyses.

SD= Standard Deviation.

IDACI=Income Deprivation Affecting Children Index.

BBSCQ=Beyond Blue School Climate Questionnaire.

a Adjusted for baseline school size, proportion of free school meals, and IDACI score, and student sex, ethnicity, family affluence score and family structure.

b Adjusted for baseline school size, proportion of free school meals, and IDACI score, and student sex, ethnicity, family affluence score, family structure, conduct & emotional problems, mental wellbeing.

These associations remained after adjustment for the core covariates (baseline measures of school size, proportion of free school meals, and IDACI score, and student sex, ethnicity, family affluence score and family structure). However, when also adjusting for baseline mental health (SDQ and SWEMWBS), these associations were markedly reduced so that, of the subscales of BBSCQ, only the associations of student commitment to learning with conduct and emotional problems, and with mental wellbeing at follow-up were significant (p ​= ​0.001 and 0.017 respectively), and the associations of these outcomes with the overall scale were of borderline significance (p ​= ​0.056 and 0.078 respectively).

For the school-level measure of positive school climate at baseline, looking at each of the subscales and overall, unadjusted analyses suggested an association between positive school climate and better student mental health outcomes at 36 months. On the overall scale, for each 1-point increase on the school BBSCQ score at baseline, conduct and emotional problems (SDQ) at follow-up months were on average 5.59 points lower (95% confidence interval (CI): −8.81 to −2.37, p-value ​= ​0.001) and mental wellbeing (SWEMWBS) was 5.61 points higher (95% CI: 1.74 to 9.48, p-value ​= ​0.004), corresponding roughly to one standard deviation, both in the direction of better mental health.

After adjustment for the covariates other than baseline mental health, these associations reduced but were not attenuated entirely. These remained statistically significant or of borderline statistical significance level (p-value from 0.013 to 0.172) for the associations between our outcomes at follow-up and most aspects of school climate at baseline, except for the commitment subscale, for which no significant association with mental health at follow-up remained. However, after also adjusting for baseline mental health, these associations reduced markedly and were no longer statistical significant.

We fitted an additional adjusted model simultaneously including school-level climate, and differential student climate score (Table 4). Similarly to earlier results, student-level and school-level climate were both significantly associated with the outcomes, after adjusting for one another and the core covariates. But these associations reduced after controlling for students’ baseline mental health. Only student-level commitment to learning remained significantly associated with conduct and emotional problems, and with mentall wellbeing (p ​= ​0.001 and 0.015, respectively).

**Table 4:** Associations between baseline student-level differential climate score and school-level measures of school climate and student mental health at 36 months.

Exposure		Outcomes											
		Conduct & emotional problems						Mental wellbeing					
		Adjusted analysis^a^			Adjusted analysis^b^			Adjusted analysis^a^			Adjusted analysis^b^		
		Coeffic.	95% CI	P	Coeffic.	95% CI	P	Coeffic.	95% CI	P	Coeffic.	95% CI	P
Student level differential climate score^c^	Teacher relationships	−1.85	−2.48 to −1.23	<0.001	−0.07	−0.55 to 0.41	0.764	1.43	0.85 to 2.02	<0.001	0.09	−0.45 to 0.62	0.741
	School belonging	−2.54	−3.22 to 1.86	<0.001	−0.34	−0.88 to 0.20	0.214	2.00	1.39 to 2.61	<0.001	0.39	−0.19 to 0.97	0.187
	Participation in decisions	−1.56	−2.15 to −0.96	<0.001	−0.28	−0.75 to 0.19	0.243	1.39	0.79 to 1.98	<0.001	0.36	−0.15 to 0.86	0.166
	Commitment to learning	−3.60	−4.58 to 2.63	<0.001	−1.32	−2.08 to −0.56	0.001	2.73	1.89 to 3.58	<0.001	0.89	0.18 to 1.61	0.015
	Overall	−3.74	−4.82 to 2.66	<0.001	−0.76	−1.57 to 0.05	0.067	2.97	2.02 to 3.92	<0.001	0.70	−0.12 to 1.52	0.092
School level BBSCQ	Teacher relationships	−2.20	−3.87 to −0.54	0.010	−1.04	−2.88 to 0.79	0.263	1.63	−0.67 to 3.93	0.165	0.90	−1.36 to 3.15	0.435
	School belonging	−2.15	−4.3 to 0.01	0.051	−0.88	−3.28 to 1.52	0.473	2.80	−0.01 to 5.61	0.051	1.97	−0.83 to 4.76	0.168
	Participation in decisions	−2.35	−4.3 to −0.4	0.018	−1.09	−3.31 to 1.13	0.334	2.51	−0.13 to 5.16	0.063	2.00	−0.57 to 4.58	0.127
	Commitment to learning	3.13	−4.11 to 10.37	0.396	3.77	−3.86 to 11.40	0.333	−6.00	−15.35 to 3.36	0.209	−4.81	−13.75 to 4.12	0.291
	Overall	−3.29	−5.91 to −0.67	0.014	−1.47	−4.44 to 1.50	0.331	3.12	−0.56 to 6.8	0.097	2.16	−1.44 to 5.75	0.240

Based on imputed dataset, N ​= ​3913 for all analyses.

IDACI=Income Deprivation Affecting Children Index.

BBSCQ=Beyond Blue School Climate Questionnaire.

a Adjusted for baseline school size, proportion of free school meals, and IDACI score, and student sex, ethnicity, family affluence score and family structure.

b Adjusted for baseline school size, proportion of free school meals, and IDACI score, and student sex, ethnicity, family affluence score, family structure, conduct & emotional problems, and mental wellbeing.

c Student BBSCQ score minus the school-mean BBSCQ score.

## 4 Discussion

### 4.1 Summary of key findings

Our results differed markedly according to whether or not our analyses adjusted for baseline mental health. In analyses adjusting for all covariates other than baseline mental health, we found that, in line with previous research (Aldridge & McChesney, 2018; England Mental Health Taskforce, 2016), we found strong and consistent associations between student-level reports of positive school climate at baseline and better mental health in terms of reduced conduct and emotional problems and better mental wellbeing at 36 months. Similarly in these analyses, as per our second hypothesis (and in contrast to the findings of Kidger et al.‘s review and some more recent studies (Dundas et al., 2014; Kidger et al., 2012)), we also found evidence of significant associations between baseline school-level measures of positive climate and better student mental health at follow-up, which were reduced but not completely attenuated by adjustment for our core covariates. Such associations might reflect our use of measures focused on aspects of mental health more likely to be associated with school climate as discussed in our introduction. However, when also adjusting for baseline measures of student mental health, these associations were reduced, to borderline significance in the case of student-level measures and to non-significance in the case of school-level climate. In a model adjusting for school climate at the individual and school levels, the measure of student-level commitment to school remained significantly associated with mental health outcomes.

These findings are perhaps not surprising given the possibility, raised in our methods section, that adjusting for baseline mental health may under-estimate the association between baseline school climate and mental health at follow-up. This is because baseline mental health, measured after students had been at the school for almost a year, may already have been influenced by school climate and so may lie on the causal pathway between school climate and subsequent student mental health. For this reason, we also reported analyses adjusting for all covariates other than baseline mental health. It may be that the true estimates of the association between baseline school- and student-level measures of school climate and student mental health outcomes at follow-up lie somewhere between those from our two adjusted models. If associations do exist between school-level climate and subsequent student mental health, these are likely to be smaller than the student-level associations. It is also possible that some of associations between baseline school climate and subsequent mental health is due to reverse causality whereby mental health at baseline influences the reporting of school climate.

Considering the theory reviewed in our introduction (Bonell et al., 2013a; Markham and Aveyard, 2003), our findings suggest that if positive school climate does benefit student mental health, this might occur primarily via an individual-level pathway involving students with good connection to school then experiencing better mental health. Our results suggest that, at the student level, student commitment to learning appears to be more associated with good mental health outcomes than other aspects of school climate. This novel finding might be explained by existing theories, which suggest the importance of student commitment to learning in the development of an individual's practical reasoning abilities which may allow them to better manage their social and emotional health (Bonell et al., 2013a; Markham and Aveyard, 2003).

### 4.2 Strengths and limitations

Our study was a large, longitudinal study using reliable measures of our exposures and outcomes which enabled us to examine the temporality of associations between student-level and school-level measures of school climate and subsequent mental health outcomes. The schools that participated were representative of state secondary schools in south-east England and our response rate among students at baseline was very high (92.8%) and at follow-up still high (85.0%). We are aware from previous analyses that attrition was higher among at-risk students so we used multiple imputation to minimise attrition bias, whereby students most likely to report negatively on school climate and their own mental health at baseline are more likely to drop out (Bonell et al., 2018). Nonetheless, it is possible that our estimates of the association between school climate (at the individual and school level) and subsequent mental health are somewhat reduced by such attrition.

Our measure of school climate identified sufficient variation between schools to enable assessments of the association of school-level climate with mental health. Of the subscales, only the subscale focused on commitment to learning was subject to limited variation between schools.

Our study was observational so any associations found cannot be assumed to be causal. As discussed in our methods section, our measure of school climate relied on student data collected near the end of their first year at secondary school. This ensured that students were able to provide information on their experiences and attitudes in relation to school climate. However, it also meant that schools climates may already have influenced student mental health, so that adjustment for baseline mental health may have over-adjusted for a variable which might plausibly lie on a causal pathway between school climate and subsequent student mental health.

It may be that measures of school-level climate that rely on aggregation of student-reported data do not measure school climate as well as objective measures. However, we think this is unlikely because students are more likely to be able to assess aspects of school climate, such as their relationships with teachers, engagement with learning and sense of active participation, than are measures based on external observations (West et al., 2004) or routine data (Aveyard et al., 2004). One option would be for measures of school climate to be based on student self-reports but from students in other year-groups so that there is less possibility of associations between school climate and mental being due to students’ baseline mental health influencing the reporting of baseline school climate. Some items of the BBSCQ focus on individual student experiences and attitudes, while others focus on broader perceptions of how the school operates. Because our focus was on measuring student-reported school climate, this was appropriate. However, the BBSCQ scale would be less suitable as a measure of individual student engagement.

### 4.3 Conclusions and implications for research

Previously, there has been evidence for associations between a positive school-level climate and health risk behaviours (such as smoking, alcohol and drug use, violence and sexual risk behaviour (Bonell et al., 2013b; Peterson et al., 2020)) but not mental health (Dundas et al., 2014; Kidger et al., 2012). Our study provides mixed evidence because of the differences in results between models. If a positive school climate does promote better student mental health, this is most likely to occur via a mechanism involving individual-level commitment to school.

Future longitudinal studies should examine the associations of school climate on subsequent mental health outcomes. Such studies should ideally measure baseline mental health prior to students entering the school, and use measures of school climate based on reports from other students in the school.

## Funding

This study was funded by the National Institute for Health Research in England under its Public Health Research Board (12/153/60) and the Education Endowment Foundation (no grant code). The views expressed in this publication are those of the authors and do not necessarily reflect those of the UK NHS, the National Institute for Health Research or the Department of Health for England.

## Role of funder/sponsor

The funder/sponsor did not participate in the work.

## Declaration of competing interest

The authors have no competing interests relevant to this article to disclose.
